# Etude histopathologique des adénopathies cervicales à Yaoundé, Cameroun

**DOI:** 10.11604/pamj.2014.19.185.4302

**Published:** 2014-10-21

**Authors:** Zacharie Sando, Florent Ymele Fouelifack, Jovanny Tsuala Fouogue, Jeanne Hortence Fouedjio, Yvette Sandrine Ngo Ndeby, Francois Djomou, Amadou Fewou, Jean-Louis Essame Oyono

**Affiliations:** 1 Service d'Anatomie Pathologique de l'Hôpital Gynéco-Obstétrique et Pédiatrique de Yaoundé, Cameroun; 2 Faculté de Médecine et des Sciences Biomédicales de l'Université de Yaoundé 1, Cameroun; 3 Hôpital Central de Yaoundé, Cameroun; 4 Service d'Oto-Rhino-Laryngologie du Centre Hospitalier et Universitaire de Yaoundé; 5 Service d'Anatomie Pathologique du Centre Hospitalier et Universitaire de Yaoundé, Cameroun

**Keywords:** Adénopathies cervicales, histopathologie, pathologie, ganglions, Yaoundé, tuberculose ganglionnaire, lymphadénite, Cameroun, cervical lymphadenopathy, histopathology, pathology, lymph nodes, Yaoundé, lymph node tuberculosis, lymphadenitis, Cameroon

## Abstract

**Introduction:**

Les adénopathies cervicales ont été très peu étudiées au Cameroun.

**Méthodes:**

Pour améliorer leur connaissance nous avons mené une étude rétrospective et descriptive dans les cinq principaux laboratoires de la capitale du pays en vue d'en déterminer les aspects histopathologiques.

**Résultats:**

Nous avons colligé 292 cas. L’âge moyen était de 30,95 ±4,11ans et le ratio homme / femme était de 0,96/1. Les principaux diagnostics histologiques étaient les suivants: tuberculose ganglionnaire (39,38%), les lymphadénites (18,49%), les lymphomes malins non hodgkiniens (12,33%), la maladie de Kaposi ganglionnaire (5,82%), les métastases de carcinome (5,82%) et la maladie de Hodgkin (5,14%). Dans 95,65% des cas le diagnostic était posé au-delà du deuxième mois d’évolution des symptômes.

**Conclusion:**

Nos résultats, quoique préliminaires, sont une contribution à l'amélioration de la stratégie diagnostique et thérapeutique dans nos hôpitaux aux ressources limitées.

## Introduction

Malgré la diversité de la pathologie ganglionnaire qui rend compte de la physiologie de ces organes lymphoïdes périphériques, elle reste dominée par les proliférations tumorales aux côtés desquelles les modifications réactionnelles issues d'une simple stimulation lymphocytaire et les réactions inflammatoires constituent de véritables sources de confusion pour le pathologiste [1, 2]. La situation de carrefour de la région cervicale rend la topographie et la pathologie de ces ganglions lymphatiques plus complexes et diverses. Ceci est d'autant plus vrai qu'on sait que le cou abrite environ 300 des 800 ganglions que comporte le corps humains [3].

Pour des raisons pratiques et dans le but de stadifier les carcinomes épidermoïdes cervico-maxillo-faciaux, les ganglions lymphatiques du cou à l'exclusion des ganglions parotidiens, sus-claviculaires et retro-pharyngés, ont été répartis en sept groupes: le groupe I (ganglions sub-mandibulaires et sous-mentaux), le groupe II (ganglions supérieurs de la chaine jugulaire interne ou groupe hyoïdien), le groupe III (ganglions moyens de la chaine jugulaire interne ou groupe supra-omohyoïdien), le groupe IV (ganglions inférieurs de la chaine jugulaire ou groupe infra-omohyoïdien), le groupe V (ganglions du triangle postérieur), le groupe VI (groupe thyroïdien) et le groupe VII (ganglions trachéo-oesophagien et médiastinaux supérieurs)[3]. Malgré d'importantes avancées dans l'imagerie ganglionnaire, l’étude histologique demeure incontournable dans le diagnostic étiologique des adénopathies cervicales. Au Cameroun aucune étude récente sur la pathologie ganglionnaire cervicale n'est disponible. C'est pour contribuer à sa meilleure connaissance que nous avons mené cette étude avec pour objectif principal de décrire les aspects histopathologiques des adénopathies cervicales observées dans la capitale du pays.

## Méthodes

L’étude était rétrospective et descriptive, sur une période de cinq ans (du 1er janvier 2005 au 31 décembre 2009). Elle a été menée dans les cinq laboratoires d'anatomie pathologique que compte la ville de Yaoundé (notamment les laboratoires du Centre Pasteur du Cameroun, de l'Hôpital Gynéco-obstétrique et Pédiatrique de Yaoundé, de l'Hôpital Général de Yaoundé, du Centre Hospitalier Universitaire, et de l'Hôpital Central de Yaoundé). Nous avons consulté les registres des laboratoires suscités à la recherche des informations suivantes pour chaque prélèvement d'adénopathie cervicale: âge, sexe du patient, site du ganglion prélevé, durée d’évolution clinique et diagnostic histologique. Etait inclu tout patient ayant bénéficié d'un prélèvement d'adénopathie cervicale au cours de la période d’étude. Etait exclu tout patient dont les informations étaient très incomplètes pour remplir la fiche technique. Ces données étaient reportées sur une fiche technique anonyme. Une clairance du comité national d’éthique a été obtenue au préalable et les données ont été analysées grâce aux logiciels Statistical Package for Social sciences (SPSS) version 10.1 et Microsoft Excel 2007.

## Résultats

Nous avons colligé 292 cas.

### Données sociodémographiques et cliniques globales

**Figure 1:**
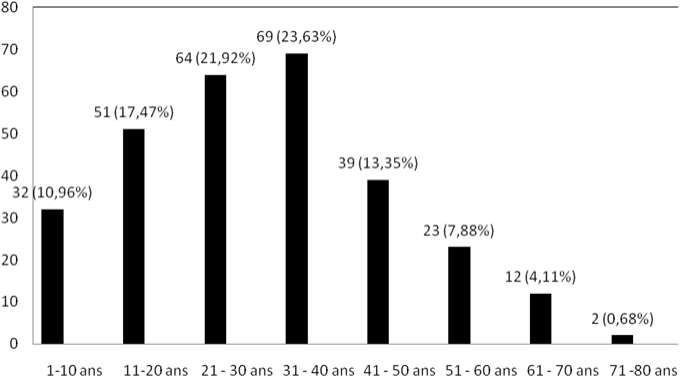
Distribution des adénopathies selon l’âge des patients

Distribution des adénopathies cervicales selon le sexe et l’âge des patients: sur 292 prélèvements, 143 étaient faits chez les sujets de sexe masculin et 149 chez les sujets chez les sujets de sexe féminin, soit un ratio homme / femme de 0,96/1. La Figure 1 montre la distribution des adénopathies selon les tranches d’âge (de 10 ans) des patients. L’âge moyen des patients était de 30,95 ± 4,11 ans avec des extrêmes allant de 1 à 79 ans. Les classes d’âge de 21 à 30 et de 31 à 40 ans étaient les plus représentées avec respectivement 64 (21,92%) et 69 (23,63%) cas.

**Figure 2:**
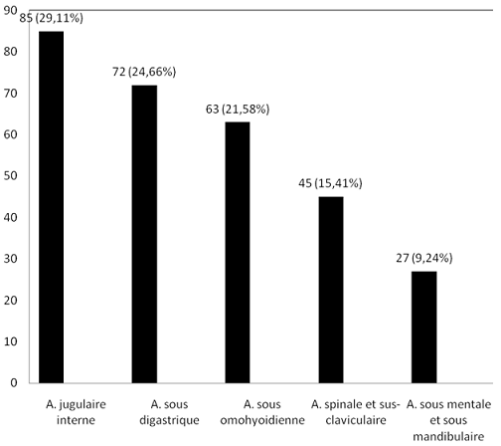
Distribution des patients selon l'aire ganglionnaire

Distribution des patients selon l'aire ganglionnaire atteinte: la Figure 2 illustre la répartition des patients en fonction des aires ganglionnaires atteintes. L'aire jugulaire interne était la plus représentée avec 85 des 292 cas (soit 29, 11%) suivie des aires sous digastrique (24,66%) et sous omohyoïdienne (21,58%).

**Figure 3:**
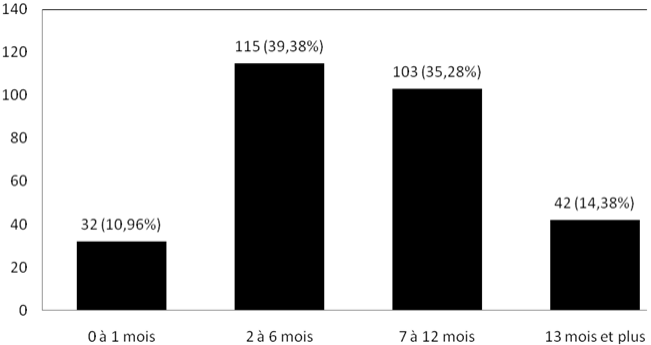
Distribution des patients en fonction de la durée d’évolution des symptoms

Distribution des patients selon la durée d’évolution des symptômes: la Figure 3 montre la distribution des patients en fonction de la durée d’évolution des symptômes. Sur 292 adénopathies, 218 (soit 74,66%) évoluaient depuis 2 à 12 mois.

### Données histopathologiques globales

Distribution des adénopathies en fonction du type histologique: le Tableau 1 représente la distribution des adénopathies en fonction du type histologique. La tuberculose ganglionnaire était le type le histologique le plus fréquent avec 115 cas sur 292 (39,38%). Elle était suivie par l'adénite réactionnelle et le lymphome malin non hodgkinien ayant chacun 36 cas (12,33%).

**Tableau 1:** Répartition des adénopathies cervicales selon le type histologique

Types histologiques	Effectifs (pourcentage)
Tuberculose ganglionnaire	115 (39,38%)
Adénite réactionnelle	36 (12,33%)
Lymphome malin non Hodgkinnien (LMNH)	36 (12,33%)
Adénite chronique	28 (9,59%)
Maladie de Kaposi ganglionnaire	17 (5,82%)
Métastase de carcinome	17 (5,82%)
Maladie de Hodgkin	15 (5,14%)
Lymphome de Burkitt	8 (2,74%)
Adénome pléomorphe	4 (1,37%)
Hémangiome capillaire	4 (1,37%)
Pseudotumeur inflammatoire	3 (1,03%)
Histoplasmose	3 (1,03%)
Cystadénolymphome	2 (0,68%)
Leucémie lymphoïde chronique	2 (0,68%)
Lymphadénopathies angio-Immunoblastiques	1 (0,34%)
Leishmaniose	1 (0,34%)
**Total**	**292 (100%)**

La tuberculose ganglionnaire était le type le histologique le plus fréquent avec 115 cas sur 292 (39,38%). Elle était suivie par l'adénite réactionnelle et le lymphome malin non hodgkinien ayant chacun 36 cas (12,33%)

### Caractéristiques socio – démographiques et cliniques des principaux types histologiques

**Figure 4:**
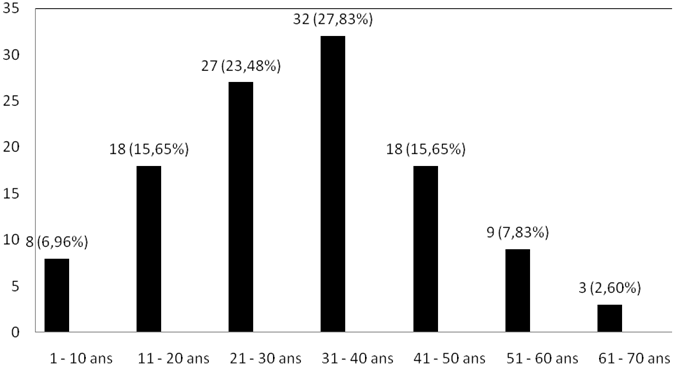
Distribution des patients atteints d'adénopathies cervicales tuberculeuses selon l’âge

**Figure 5:**
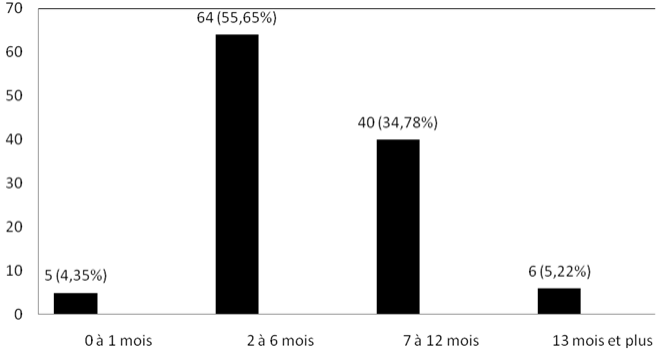
Distribution des patients atteints d'adénopathies cervicales tuberculeuses en fonction de la durée d’évolution des symptômes

**Tuberculose ganglionnaire:** sur 115 patients atteints de tuberculose ganglionnaire, 57 étaient de sexe masculin soit une sex ratio Homme/femme de 0,98/1. La Figure 4 illustre la répartition des adénopathies cervicales tuberculeuses selon l’âge des patients. La moyenne d’âge des patients atteints d'adénopathies tuberculeuses était de 31,85 ± 5,02 ans. La fréquence maximale (32 cas sur 115 soit 27,83%) des adénopathies tuberculeuses s'observait entre 31 et 40 ans. Seuls 3 cas sur 115 (soit 2,6%) étaient observés après 60 ans. La Figure 5 montre la distribution des patients atteints d'adénopathies tuberculeuses selon la durée des symptômes. Sur 115 adénopathies cervicales tuberculeuses, 69 (soit 60%) étaient diagnostiqués après au plus six mois d’évolution des symptômes.

**Autres types histologiques:** les distributions des autres types histologiques d'adénopathies selon le sexe et selon l’âge des patients sont présentées respectivement dans le Tableau 2, Tableau 3. Sur adénopathies dues au lymphome de Burkitt, 8 (soit 75%) étaient observés chez des malades de moins de10 ans. Treize des quinze cas (86,67%) d'adénopathies dues à la maladie de Hodgkin étaient observées chez des malades de moins 30 ans. Le Tableau 4 représente la répartition des adénopathies des différents types histologiques selon la durée d’évolution des symptômes. Sur 157 adénopathies 109 (soit 69,42%) étaient observées après deux à douze mois d’évolution des symptômes.

**Tableau 2:** Répartition des principaux types histologiques selon le sexe des malades

Types histologiques	Effectif		*Sex ratio*
	Sexe masculin	Sexe féminin	
Adénite réactionnelle	12	24	0,5
Lymphome malin non hodgkinnien (LMNH)	22	14	1,57
Adénite chronique	13	15	0,87
Kaposi ganglionnaire	9	8	1,13
Métastase de carcinome	15	2	7,5
Maladie de Hogdkin	8	7	1,14
Lymphome de Burkitt	6	2	3

Les *sex ratio* des métastases de carcinomes, du lymphome de Burkitt et de l'adénite réactionnelle était respectivement de 7,5; 3 et 0,5

**Tableau 3:** Distribution des autres types histologiques d'adénopathies selon l’âge des malades

Types Histologiques	1–10 ans	11 -20 ans	21-30 ans	31-40 ans	41-50 ans	51-60 ans	61-70 ans	71-80 ans	Total
Adénite réactionnelle	5 (3,18%)	10 (6,37%)	7 (4,46%)	7(4,46%)	5 (3,18%)	1 (0,64%)	0	1(0,64%)	36 (22,93%)
LMNH	1 (0,64%)	7 (4,46%)	9 (5,73%)	8 (5,09%)	3 (1,91%)	3 (1,91%)	5 (3,18%)	0	36 (22,93%)
Adénite chronique	5(3,18%)	5(3,18%)	6(3,82%)	6(3,82%)	1(0,64%)	3(1,91%)	1(0,64%)	0	28 (17,83%)
Kaposi ganglionnaire	2(1,27%)	1 (0,64%)	2 (1,27%)	5 (3,18%)	6 3,82%)	1 (0,64%)	0	0	17 (10,83%)
Métastase de Carcinome	0	1 (0,64%)	3 (3,18%)	3 (3,18%)	3 (3,18%)	4 (2,55%)	2 (1,27%)	1 (0,64%)	17 (10,83%)
Maladie de Hogdkin	2 (1,27%)	6 (3,82%)	5 (3,18%)	0	0	2 (1,27%)	0	0	15 (9,55%)
Lymphome de Burkitt	6 (3,82%)	1 (0,64%)	1 (0,64%)	0	0	0	0	0	8 (5,1%)
Total	21(13,38%)	31 (19,75%)	33 (21,02%)	29 (18,47%)	18 (11,46%)	15(9,55%)	8 (5,1%)	2 (1,27%)	157 (100%)

LMNH: Lymphome Malin Non Hodgkinien

Sur adénopathies dues au lymphome de Burkitt, 8 (soit 75%) étaient observés chez des malades de moins de10 ans. Treize des quinze cas (86,67%) d'adénopathies dues à la maladie de Hodgkin étaient observées chez des malades de moins 30 ans

**Tableau 4:** Distribution des adénopathies des différents types histologiques selon la durée d’évolution des symptômes

Types Histologiques/ durée des symptômes (mois)	0 - 1 mois	2 - 6 mois	7 – 12 mois	13 mois et plus	Total
Adénite réactionnelle	15 (9,55%)	14 (8.92%)	6 (3,82%)	1 (0,64%)	36 (22,93)
LMNH	2 (1,27%)	8 (5,1%)	15 (9,55%)	11 (701%)	36 (22,93%)
Adénite chronique	2 (1,27%)	17 (10,83%)	3 (1,91%)	6 (3,82%)	28 (17,83%)
Kaposi ganglionnaire	1 (0,64%)	1 (0,64%)	13 (8,28%)	2 (1,27%)	17 (10,83%)
Métastase de Carcinome	1 (0,64%)	6 (3,82%)	8 (5,1%)	2 (1,27%)	17 (10,83%)
Maladie de Hogdkin	2 (1,27%)	6 (3,82%)	5 (3,19%)	2 (1,27%)	15 (9,55%)
Lymphome de Burkitt	0	4 (2,55%)	3 (1,91%)	1 (0,64%)	8 (5,1%)
**Total**	**23 (14,65%)**	**56 (35,67%)**	**53 (33,76%)**	**25 (15,92%)**	**157 (100%)**

LMNH: Lymphome Malin Non Hodgkinien

Sur 157 adénopathies 109 (soit 69,42%) étaient observées après deux à douze mois d’évolution des symptômes

## Discussion

### Caractéristiques socio-démographiques et cliniques

L’âge moyen des patients de notre série était de 30,95± 4,11 ans. Globalement toutes les tranches d’âge jusqu’à 60 ans étaient concernées par les adénopathies cervicales comme le montre la Figure 1. Cependant les adultes des classes d’âge de 20 à 29 ans, 30 à 39 ans, et 40 à 49 ans constituaient 58,9% des cas. La répartition selon l’âge des patients de notre série est voisine de celle rapportée à dans la même période et deux décennies plus tôt en Afrique sub-saharienne [1, 4]. La même tendance a été rapportée par des études sur des populations asiatiques et européennes [5, 6]. Il semble que les adénopathies cervicales soient l'apanage de l'adulte jeune. La répartition des adénopathies cervicales tuberculeuses de notre série est similaire à celle rapportée par plusieurs auteurs [4, 6, 7]. Six des huit cas (75%) des lymphomes de Burkitt ont été observés chez des enfants de moins de 10 ans. Cette observation était attendue dans notre contexte car il a été rapporté qu'en Afrique Centrale le lymphome de Burkitt était plus fréquent chez l'enfant en raison de la forte prévalence de l'infection par le virus d'Epstein-Barr [8]. Sur 15 cas de maladie de Hodgkin, 8 étaient observés chez des individus de moins de 20 ans. Dans la littérature 2 pics d'incidence de la maladie de Hodgkin ont été décrits: le premier autour de 20 ans comme dans notre étude et le second autour de 80 ans [8, 9].

Sur les 292 patients observés, l'analyse histologique était réalisée après 2 à 12 mois d’évolution des symptômes chez 218 (soit 74,68%) (Figure 3). L'absence de préjudice esthétique ou fonctionnel grave immédiat causée par la plupart de adénopathies cervicales, le caractère chronique des pathologies identifiés, la pauvreté et la tendance qu'ont trois quarts des populations d'Afrique noire à recourir premièrement à la médecine traditionnelle peuvent expliquer ce retard au diagnostic [10, 11]. D'autres auteurs ont rapporté ce retard dans le diagnostic des cancers au Cameroun [12]. Dans notre étude, le sex ratio hommes /femmes était de 0,95/1. Ce ratio est proche de ceux rapportés par Ndongo et al à Dakar (Sénégal) [4] et par Ndjolo et al à Yaoundé (Cameroun)[13]. En dehors du groupe I (ganglions sous-mentaux et sous mandibulaires) qui regroupait 9,27% des cas, les groupes ganglionnaires étaient uniformément atteints avec des proportions variant de 15,41% à 29,11% (Figure 2). Ces derniers groupes sont ceux qui drainent la plupart des viscères de la région cervicale, leur atteinte s'explique par la présence des métastases carcinomateuses des organes précis qui, contrairement aux pathologies telles que la tuberculose intéressent préférentiellement certains groupes ganglionnaires [3].

### Les différents types histologiques

Les ganglions lymphatiques représentent la localisation la plus fréquente de la tuberculose extra-pulmonaire [14]. Dans notre étude les adénopathies cervicales tuberculeuses constituaient 39,38% des cas observés. Fakhry et al. et [15] Maharjan et al [16] ont respectivement obtenu 33% en Côte d'Ivoire et 54% à Kathmandou. Par contre Ndongo et al. ont rapporté 64,6% au Sénégal [4]. Ces différences s'expliquent en partie par la plus grande taille de notre échantillon (115 cas étudiés contre 33 et 31 cas) et par l'ancienneté des autres études qui datent de 5 ans [16], 31 ans [15] et 17 ans [4]. Nous avons observé que la classe d’âge modale était celle de 31 à 40 ans et que l‘âge moyen était de 31,85+/-5,02 ans. De plus, 66,96% des cas ont été observés entre 21 et 50 ans. Ceci est proche des résultats rapportés dans la littérature qui fait état des moyennes d’âge de 40 et 46 ans [6]et de proportions de 45,45% [4] à 69% [6] pour les adultes jeunes. Les lymphomes malins non hodgkiniens (LMNH) sont les tumeurs solides du système immunitaire ayant pour caractéristique commune l'absence de la cellule de Reed-Sternberg [8]. Les différences entre les sous types de LMNH réside dans leurs propriétés moléculaires que seules des techniques de biologie de pointes permettent de mettre en évidence [17].

Notre contexte limité en ressources ne permet pas à nos pathologistes d'en faire la distinction, c'est pourquoi dans ce travail nous ne parlerons que du groupe de LMNH. Leur incidence est élevée tant dans les pays riches qu'au Cameroun ù ils arrivent en tête des étiologies de adénopathies cervicales malignes [8, 13] et des cancers hématologiques chez les personnes vivant avec le VIH/SIDA [18]. Tout comme dans la série de Ndongo et al [1] les LMNH constituaient la deuxième cause des adénopathies cervicales mais les proportions diffèrent: 12,33%(n = 115 dans notre étude) contre 27% (n = 66). Les LMH sont également connus pour être plus fréquents chez les hommes que chez les femmes, et nos résultats l'ont confirmé avec un ratio hommes / femmes de 22/14 soit 1,57 [8].

La maladie de Hodgkin représentait 5,14% des cas de notre étude. Cette pathologie reste l'apanage des hommes âgés [8]. Dans notre étude les hommes étaient presqu'autant atteints que les femmes (ratio hommes / femmes de 8/7) et les sujets étaient plutôt jeunes, treize participants sur les quinze observés ayant moins de trente ans au moment du diagnostic. La faible taille de notre échantillon ne nous permettant pas de conclure. Concernant le lymphome de Burkitt, seuls 8 des 292 cas (soit 2,74%) d'adénopathies cervicales étudiées étaient causés par le lymphome de Burkitt avec une nette prédominance du sexe masculin (ratio hommes / femmes de 3/1) et une prédilection pour l'enfant: 6 cas sur 8 observés chez des sujets de moins de 10 ans. La rareté de ce type particulier de lymphome non hodgkinien tout comme sa prédilection pour les enfants sont bien connues [8, 18–20]. Nous avons retrouvé une nette prédominance pour le sexe masculin comme l'avaient fait Ndoumbé et al. en 1997 à Yaoundé (ratio hommes / femmes de 16,34/1 [19].

Le sarcome de Kaposi représentait 17 cas sur 292. Cette affection décrite pour la première fois par Moritz Kaposi Kohn à Vienne en 1872 était la cause de 5,82% des adénopathies de notre étude [21, 22]. La prédilection pour le sexe masculin et pour les enfants n'a pas été observée dans notre étude contrairement aux données de la littérature [21, 23].

Les adénites représentaient 64 cas 292 soit 21,92% dans notre étude. Les adénites réactionnelles comptaient pour 36 cas soit 12,33%, proportion inférieure à celle de 33% rapportée par Maharjan en Inde [16]. Les adénites chroniques représentaient 28 cas soit 9,59% des notre échantillon. Nous n'avons pas observé d'aspect nécrotique décrit dans les adénites de Fujimoto – Kikuchi [24]. La réalisation d'une analyse bactériologique pour chacune de nos adénopathies aurait certes accru la pertinence de notre travail mais elle ne rentrait pas dans nos objectifs. En effet les infections bactériennes sont des causes très fréquentes d'adénopathies [25, 26].

## Conclusion

Les adénopathies cervicales concernent toutes les classes d’âges et sont dominées par la tuberculose, la pathologie tumorale et inflammatoire. Le diagnostic est très souvent tardif. Une meilleure connaissance de leurs aspects histologiques usuels permettrait de réorienter les stratégies de prévention et de prise en charge dans notre système de santé aux ressources limitées.
